# Characterizing the effects of structural fires on fine particulate matter with a dense sensing network

**DOI:** 10.1038/s41598-023-38392-3

**Published:** 2023-08-08

**Authors:** Ayina Anyachebelu, Alex Cabral, Marah I. Abdin, Pallavi Choudhury, Madeleine I. G. Daepp

**Affiliations:** 1 Department of Civil, Environmental and Geomatic Engineering, University College London, London, WC1E 7HB UK; 2 John A. Paulson School of Engineering and Applied Sciences, Harvard University, Boston, MA 02134 USA; 3 Microsoft Research, Redmond, WA 98052 USA

**Keywords:** Environmental social sciences, Environmental impact

## Abstract

Short-term increases in air pollution levels are linked to large adverse effects on health and productivity. However, existing regulatory monitoring systems lack the spatial or temporal resolution needed to capture localized events. This study uses a dense network of over 100 sensors, deployed across the city of Chicago, Illinois, to capture the spread of smoke from short-term structural fire events. Examining all large structural fires that occurred in the city over a year (N = 21), we characterize differences in PM$_{2.5}$ concentrations downwind versus upwind of the fires. On average, we observed increases of up to 10.7 $\upmu$g/m$^{3}$ (95% CI 5.7–15.7) for sensors within 2 km and up to 7.7 $\upmu$g/m$^{3}$ (95% CI 3.4–12.0) for sensors 2–5 km downwind of fires. Statistically significant elevated concentrations were evident as far as 5 km downwind of the location of the fire and persisted over approximately 2 h on average. This work shows how low-cost sensors can provide insight on local and short-term pollution events, enabling regulators to provide timely warnings to vulnerable populations.

## Introduction

Air pollution is the leading environmental risk factor for morbidity and mortality globally^1^. All but 1% of the world’s population breathes air exceeding World Health Organization air quality limits^2^, with inequitable exposures implicated in disparities in respiratory and cardiovascular disease^3,4^, adverse pregnancy outcomes^5,6^, and other morbidities^7,8^. Less is known, however, about the contributions of short-term and localized events to air pollution burdens.

Short-term ambient air pollution events adversely impact health^9–11^ as well as productivity^12^. Even small increases in fine particulate matter (PM$_{2.5}$) concentrations over periods as short as 2 hours are associated with increased heart attack and stroke risk^13^. Short-term and localized exposures may also be more easily mitigated than chronic exposures, for example via timely warning systems that lead people to close windows to prevent indoor infiltration^14^ and to postpone outdoor activities^15^. However, transient and local events are often undetected by regulatory monitors, which are sparsely distributed across cities^16^. Many regulatory monitoring systems also collect data infrequently or at coarse temporal resolutions, missing episodic emissions^17^, and extensive quality assurance processes can introduce delays between when an event occurs and when the public learns of its effects. A recent proliferation of low-cost air quality sensing networks promises to address these challenges, but existing research has tended to focus on monitoring sources known a priori in order to support local advocacy agendas^18,19^ or on generalized anomaly detection^20–22^, rather than on capturing unpredictable events. This paper seeks to fill this research gap by showing how a large-scale, low-cost sensor network can characterize the changes in PM$_{2.5}$ concentrations associated with structural fires in a major city over a full year of observation.

Structural fires—fires involving the structural component of various types of residential, commercial, or industrial buildings—are an important yet under-monitored cause of short-term and localized pollution episodes. In the United States, there are approximately half a million structural fires every year^23^. But structural fires are unpredictable events that rarely occur immediately upwind of a regulatory monitor, and thus the few studies seeking to better quantify their impacts have focused on major industrial accidents^24–26^. To our knowledge, there is no research on increases in PM$_{2.5}$ concentrations associated with general structural fires that, although smaller, are far more frequent^23^. Moreover, although an extensive literature has documented socioeconomic and racial inequities in the locations of fires as well as in their contributions to property damages, injury, and mortality^27,28^, little research has examined inequities in their effects on pollution—which may considerably magnify health consequences, particularly if the nearby population is affected by pre-existing health vulnerabilities.

In this paper, we show how a network of low-cost sensors can fill key monitoring gaps. We characterize effects on PM$_{2.5}$, which is one of many pollutants that fires emit, for three reasons: (1) PM$_{2.5}$ has been shown to affect health even at low levels^7,29^; (2) the PM$_{2.5}$ from fires may be particularly health-hazardous because it contains trace metals and other harmful byproducts of the burning of synthetic materials^30,31^; and (3) innovations in optical particle sensing and sensor calibration increasingly enable reliable low-cost measurement of PM$_{2.5}$ concentrations^32^, making it possible for us to deploy a network with frequent readings and spatially dense monitoring^33^. We combine in-situ PM$_{2.5}$ observations with meteorological observations of wind direction to compare concentrations downwind versus upwind of structural fires, applying a difference-in-differences approach to characterize the average time period over which smoke persists. Our findings demonstrate the promise of low-cost sensor networks to characterize health-relevant, hyperlocal changes in PM$_{2.5}$ that routine environmental monitoring systems have previously struggled to detect.

## Data

To obtain air quality data, we deployed a network of 115 low-cost wireless sensing nodes across Chicago bus shelters. Named Project Eclipse, the initiative was a collaboration with partners including the Chicago Department of Public Health and JCDecaux Chicago, the local affiliate of the global advertising agency JCDecaux SA, the world’s largest provider of outdoor street furniture. The original goal of the network was twofold: first, to provide citywide coverage that could support the data needs of city and academic researchers; and second, to provide additional monitoring in environmental justice neighborhoods where residents have historically been underserved by environmental monitoring^33^. The resulting network was more spatially dense than existing routine environmental monitoring systems: the average Chicago resident lived within 0.65 miles of one of our sensing nodes compared to 1.6 miles and 3.3 miles from crowd-sourced and regulatory monitors, respectively^33^.

Briefly, Eclipse devices report measurements of PM$_{2.5}$, collected every 5 min using a Sensirion SPS30 optical particle sensor, as well as temperature, relative humidity, pressure, and a set of four gaseous pollutants. We deployed devices starting in July 2021. We determined locations using a three-step framework: first, we selected 80 sites identified using a stratified random sampling design following Matte et al.^34^; second, we worked with community groups and local partners to select 26 additional sites in environmental justice areas; and finally, we co-located 3 additional devices with each of three regulatory monitoring stations (n = 9 devices total). Because low-cost optical particle sensors can be subject to error, the research team used these co-located devices to develop a calibration algorithm that improved accuracy to levels consistent with EPA recommendations for low-cost sensors^35^. We have included a detailed description of the calibration method and results in Appendix A of the supplementary information. For further details on the network design and hardware, please see Daepp et al.^36^; the calibrated data and further details on the calibration method are publicly available^37^.

To identify structural fires, we collected all fire reports posted to the City of Chicago Fire Department’s public Twitter page between July 1, 2021 and June 30, 2022^38^. Posts include each fire’s location (street address), start time, and alarm level—a rating from 1 to 3 indicating the amount of units and firefighters needed to contain the fire, where 3-alarm fires were the largest observed in Chicago during the study period. Although the listing of 1-alarm fires was not comprehensive, the Fire Department Media team confirmed that the list included all 2- and 3-alarm fires in the study period; for the purpose of this paper, we thus constrain our analyses to the multi-alarm fires. To ensure the accuracy of each variable, we further cross-referenced the data against local news reports for each fire. We then geocoded locations using Nominatim (OpenStreetMap)^39^.

**Figure 1:**
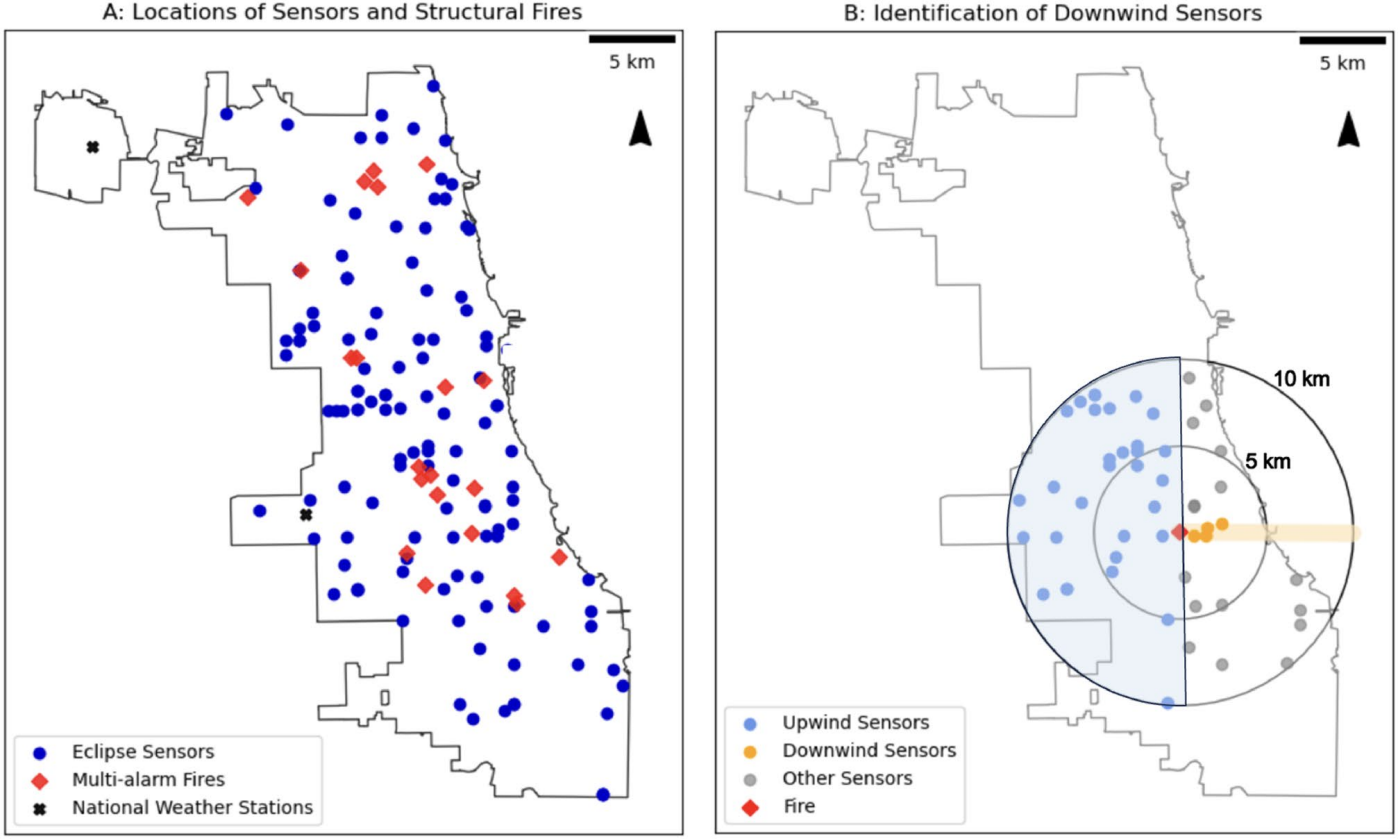
Panel (**A**): Locations of Eclipse sensors (blue points), multi-alarm fires (red diamonds), and NOAA Weather Stations (black crosses) within the entire city limits of Chicago. Panel (**B**): Identification of Downwind and Upwind Sensors For One Fire. Black circles are radii of 5 km and 10 km from the fire. Sensors falling within these circles are used in the analysis. The rectangular orange band represents the fire emissions path in direction of the wind (east). Sensors that fall into the band (orange points) are labelled as downwind, sensors that fall into the highlighted semicircle (blue points) are upwind, and all other sensors (gray points) are not included in the analysis.

Finally, we obtained meteorology data from the National Oceanic and Atmospheric Administration (NOAA) via the Meteostat weather database^40^, which included data from two NOAA weather stations in Chicago (Figure 1 Panel A). We linked each sensor reading with the wind direction, wind speed, temperature, and precipitation of the weather station closest to the corresponding fire.

Our raw structural fire data set includes 23 multi-alarm fires from July 2021 to July 2022. We further remove 2 fires that occurred at a time with no dominant wind direction. For the remaining 21 multi-alarm fires, we obtain 152,275 PM$_{2.5}$ readings from all sensors during the 3 h before and after the fires. Following Lu et al.^41^, we apply a three-step quality assurance/control procedure. First, we exclude PM$_{2.5}$ outliers with abnormal 5-min values equal to or less than ${0}\upmu \hbox {g}/\hbox {m}^3$ or greater than ${1000}\upmu \hbox {g}/\hbox {m}^3$ (0.01%) to mitigate the effects of sensor malfunctions. Second, to further ensure the exclusion of malfunctioning devices, we remove sensors with less than 75% of the 73 readings expected during the 6-hour monitoring period of each fire (1.19%). Finally, to account for skipped readings, we impute missing readings within a given sensor using linear interpolation. Our final data set has 156,667 5-min readings for the 3 h before and after the 21 fires.

## Methods

We use a difference-in-differences estimation approach to evaluate the effects of structural fires on PM$_{2.5}$ readings, comparing concentrations observed using sensors downwind versus upwind of each fire after versus before the fire’s start. This approach exploits the role of wind direction, which dictates the local transport of pollutants^9,42,43^, to control for potential sources of confounding variables. Fires may, for example, be more likely to occur in neighborhoods that also have higher baseline levels of PM$_{2.5}$; however, both the downwind and upwind sensors would be similarly affected, and thus the upwind sensors would act as a control for the relatively higher readings that would have been expected even in the absence of a fire. Similarly, a citywide pollution event could coincide with the start of a fire—but the increase in concentration would be observed in the upwind as well as the downwind sensors, and thus would not affect the estimated difference. The identifying assumption is thus that any factors besides the fires that contribute to short-term changes in PM$_{2.5}$ (including other local pollution sources or gradients of other pollutants that influence secondary organic aerosol production) are not consistently either upwind or downwind of fires but rather distributed at random, and thus do not bias the difference-in-differences estimator across the population of fires.

### Identifying downwind and upwind sensors

Given the limited literature on fire plumes in urban areas, we choose to create a simplistic version of a plume that can generalize to the 21 structural fires included in the analyses. Using the average building height and road width in Chicago^44,45^, we determine that the average street has a medium ratio of road width to building height, thus resulting in moderate flushing rates of PM$_{2.5}$ based on the air flow and potential to concentrate locally-emitted pollution^46^. As a result, we use a wide rectangular band in the direction of the fire (Fig. 1 Panel B) to represent the fire plume—wide to acknowledge the dispersion on roads that are not street canyons, and rectangular to accommodate the air flow patterns. We set the width of the band to 1 km in the primary analysis, a distance that enables the inclusion of more downwind sensors in comparison to smaller widths but excludes unaffected sensors indicated by the similar but diminishing effect sizes with wider bands in robustness tests (see Results for further details).

To determine the control group–hereafter referred to as upwind sensors—we consider any sensors at least a 90 degree angle away from the wind direction as upwind. That is, sensors are classified as “upwind” of a given fire if they are located within the semi-circle in the opposite direction of the prevailing wind, as shown in Fig. 1. We consider any sensors in the downwind band as downwind. As a result, a sensor can be considered as a downwind sensor for one fire and upwind sensor for a different fire. We examine the association of measurements with distance from the fire by calculating bands of lengths 2, 5 and 10 km. To ensure that the upwind sensors reflect a comparable control group (e.g. for neighborhood-specific concentrations levels) to the downwind sensors, we restrict our analysis to sensors that are within 5 or 10 km of fires.

For all 21 multi-alarm fires that are used in the analyses, 16 (76%) have at least one downwind sensor within 10 km and 14 (67%) have a downwind sensor within 5 km. On average, each fire has 2.1 and 2.5 downwind sensors within 5 km and 10 km respectively.

**Figure 2:**
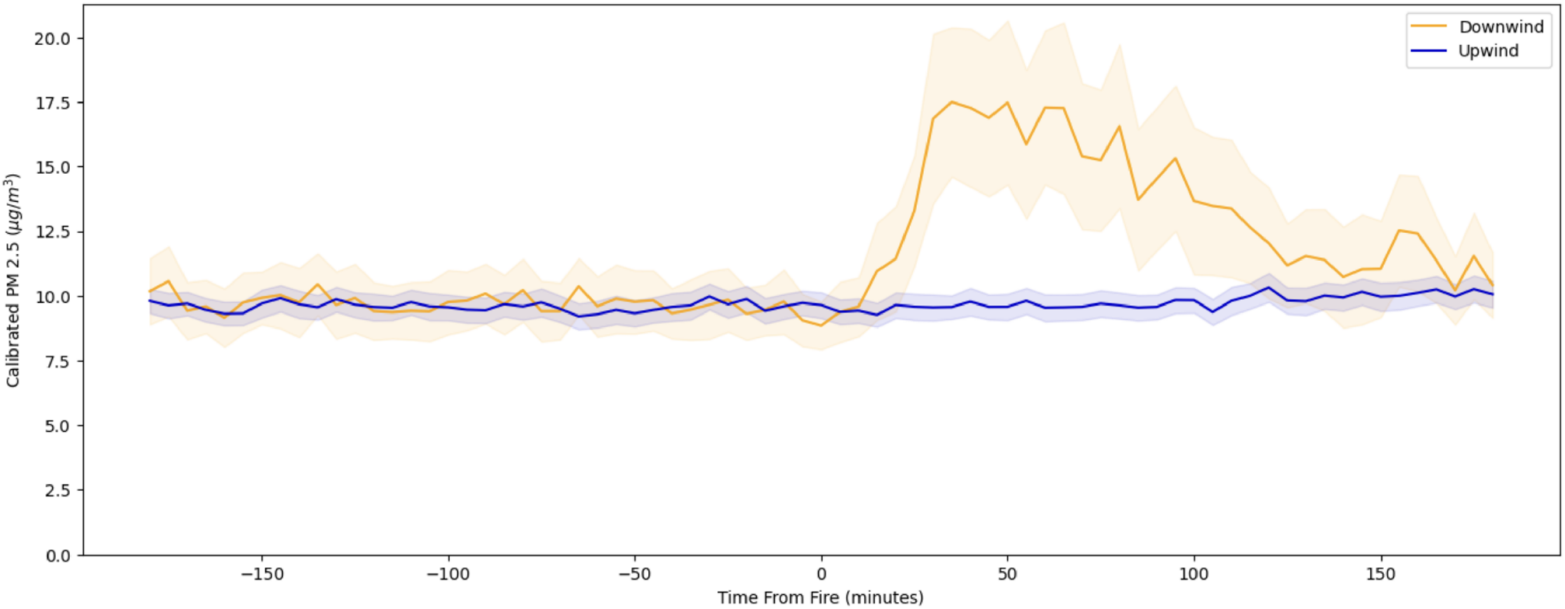
Parallel Trends Plot: Average calibrated PM$_{2.5}$ readings for downwind and upwind sensors before and after fires. Lower and upper bounds of confidence intervals are 2 standard errors from the mean. All sensors included in the figure are within 5 km of fires. Number of downwind sensors = 29. Number of upwind sensors = 312.

Before conducting our main analyses, we compared the trends in PM$_{2.5}$ readings *before* the start of the fires. The difference-in-difference approach relies on the identifying assumption that the treatment and control groups (downwind and upwind sensors) would have followed parallel trends in the absence of a fire. Figure 2 plots the trends for the average 5-min readings of PM$_{2.5}$ for the sensor groups during the 3 h before and after the fires (Fig. 2). Although we cannot test the assumption, evidence of parallel trends in the pre-period—before the fire—bolsters confidence that the assumption holds.

### Model

We estimate the relationship between structural fires and calibrated PM$_{2.5}$ for sensor *i* at time *t* using the model:

1 $$\begin{aligned} PM_{it} = \alpha + \sum \limits _{k = - 35}^{ - 1} \beta _{{\text {k}}} Downwind _{it}^{k} + \sum \limits _{k = 1}^{35} \beta _{{\text {k}}} Downwind _{it}^{k} + X'_{it}\Gamma + \mu _{i} + \lambda _{t} + \varepsilon _{it} \end{aligned}$$

We include a set of time-variant dummy variables, $Downwind _{it}^{k}$, to allow for a non-parametric period-specific effect of the fire. *k* ranges from $-35$ to 35 which indexes the 35 5-mi readings for the 180 min (3 h) before and after the fires. For each sensor, we have an equal number of readings before and after the fire, ensuring balanced data in our pre- and post- fire periods. The coefficients of interest, $\beta _{{\text {k}}}$, denote the difference in PM$_{2.5}$ between downwind and upwind sensors in the 5$k_{th}$ minute. This approach enables us to examine how the effect changes over time. In addition to offering insight on the peak and duration of effects, we note that the lack of any observable trend in the resulting coefficients prior to the fire would offer further evidence of parallel trends in the pre-period.

To investigate how the effect on PM$_{2.5}$ varies over space, we also stratify the treatment group based on distance (0–2 km, 2–5 km, and 5–10 km) from the fire. We choose these distance bins to ensure that there are enough downwind sensors in each distance group for analysis. To control for sensor-specific factors, diurnal or seasonal variation, and fire-specific differences, our specification includes $\mu _{i}$, a sensor-specific fixed effect, as well as $\lambda _{t}$, denoting time (month, hour and fire) fixed effects. Additionally, we run the regression including a vector of meteorological parameters, X’$_{it}$, including wind speed, temperature and total precipitation in the 24 h before the fire, given that several studies show that these meteorological factors influence PM$_{2.5}$ concentrations^43,47–49^.

Finally, to examine inequities in the PM$_{2.5}$ burdens associated with fires, we estimate the average demographic makeup of areas affected by fire plumes relative to the demographic makeup of the city as a whole. For each fire, we identify all census block groups that overlap with the 5 km downwind bands; we then evaluate differences in the demographic characteristics (obtained from the 2016-2020 5-year American Community Survey) for affected versus unaffected areas^50^.

We conduct several sensitivity analyses to probe the robustness of our results. Across models, we cluster standard errors at the sensor level; as an additional robustness check, we also fit models with clustering at the fire level to account for possible serial correlation within a fire. Because our data are derived from low-cost sensors—meaning that the estimated effect magnitudes may be subject to measurement error–we also fit the specification using the natural log of the outcome variable to observe relative effects. Finally, we rerun the analysis using a greater selection of upwind sensors that includes all sensors within 5 km that do not fall into the rectangular downwind band, to evaluate the robustness of our analysis to the way in which we identify upwind sensors. We further check the sensitivity of our results to our definition of downwind sensors by fitting models with several different widths for the rectangular downwind band.

## Results

**Figure 3:**
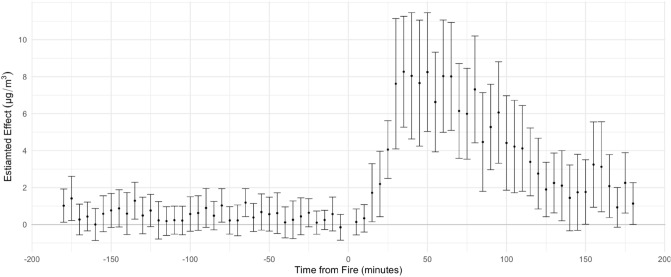
Model-estimated effect of structural fires on PM_2.5_ Levels Using 1000 m wide and 5 km long downwind band. All sensors are within 5 km of the fire. Standard errors are clustered at the sensor level. Estimates are from model including time, fire-specific, and sensor-specific fixed effects and meteorological parameters. Number of Fires = 21. Number of downwind sensors = 29. Number of upwind sensors = 158.

Figure 3 shows the estimated effect of multi-alarm structural fires on PM$_{2.5}$ over time. Comparing sensors that are downwind versus upwind and restricting our analysis to sensors within 5 km of the fire’s location, we observe statistically significant differences after but not before the fire’s start.

Overall, sensors had an average reading of 10.2 $\upmu$g/m$^{3}$ (SD = 3.8) for the hour before the start of fires. The estimated difference in PM$_{2.5}$ readings of downwind versus upwind sensors increases after a fire’s start. On average, PM$_{2.5}$ levels for downwind sensors begin increasing 15 min after the start of the fire and remain elevated for over 2 hours. The average peak, or largest difference, is a 8.3 $\upmu$g/m$^{3}$ 95% CI [5.2–11.2] increase in PM$_{2.5}$ that is observed approximately 35 min after the fire’s reported start time. In general, PM$_{2.5}$ readings are approximately over 5 $\upmu$g/m$^{3}$ higher than the baseline from 30 to 100 min after the fire.

Effects are robust to a larger selection of upwind sensors and to the use of a range of different widths for the rectangular band identifying downwind sensors, as well as to the inclusion of fire fixed effects, and to two-way clustering of standard errors by fire and sensor (See Supplementary Information). We report results for the robustness checks in the Supplementary Information. We further note that the pre-fire coefficients from Fig. 3 are stable over time and not significantly different from 0, again bolstering our confidence with respect to the parallel trends assumption.

We next investigate the average spatial extent of the changes in PM$_{2.5}$. We stratify sensors by distance from each fire (Fig. 5). As expected, the observed increase in concentrations is smaller for sensors further from a given fire. Statistically significant increased readings are observed at downwind sensors 0–2 km and 2–5 km from fires, but not for sensors more than 5km away. Sensors within 2 km had increases as high as 10.7 $\upmu$g/m$^{3}$ 95% CI [5.7–15.7]. Effects 2–5 km away are similarly persistent, albeit with smaller peaks corresponding to 7.7 $\upmu$g/m$^{3}$ 95% CI [3.4–12.0] increases in concentrations.

While the model estimates the effect on concentrations across all fires, the impact of individual fires varied. We observed differences in PM$_{2.5}$ as high as over 20 $\upmu$g/m$^{3}$ between the raw readings of downwind and upwind sensors after a singular fire. The average readings of downwind sensors for the individual fires can be found in the Supplementary Information.

**Figure 4:**
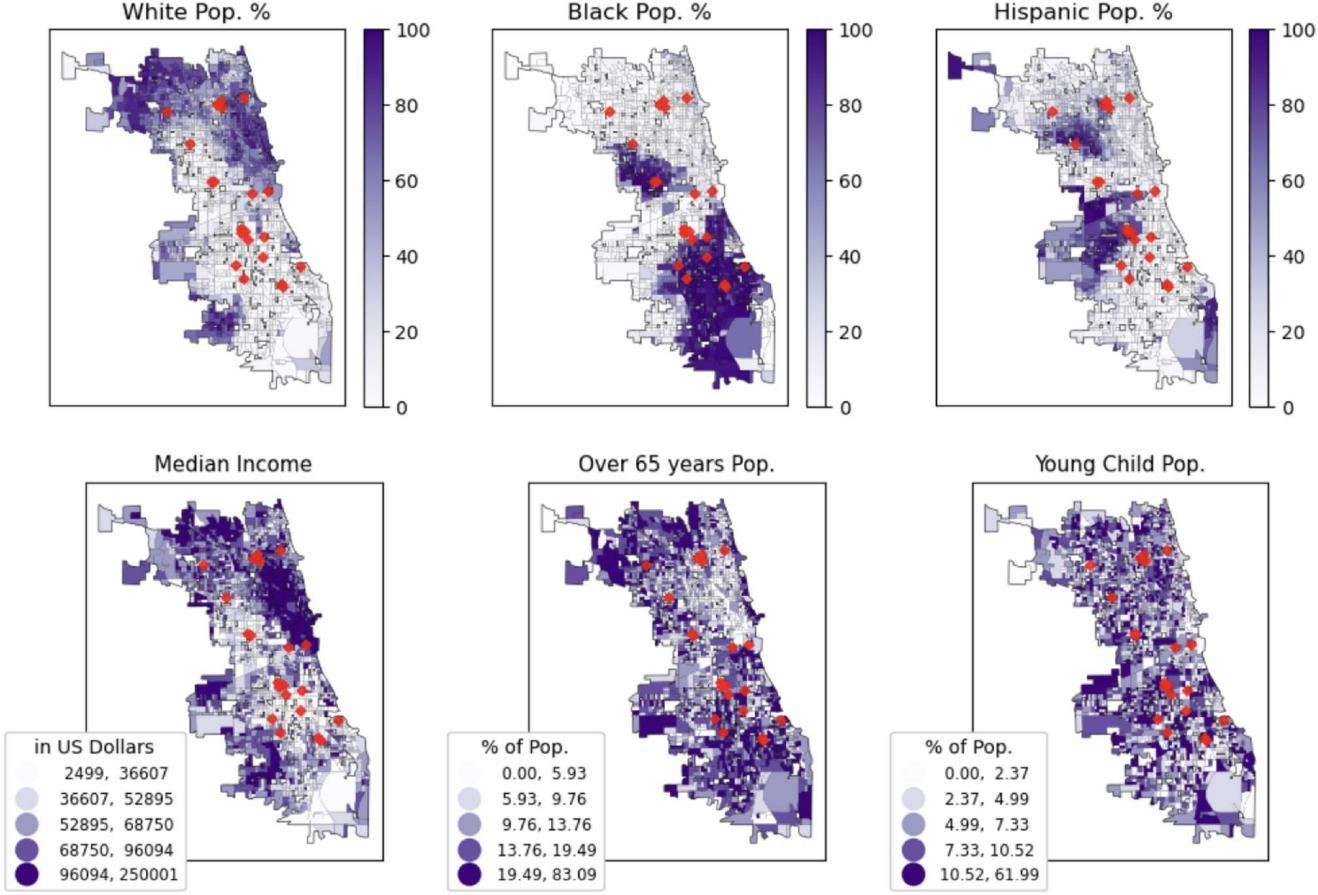
Locations of multi-alarm structural fires (red diamonds) and socioeconomic or demographic makeup in Chicago.

**Figure 5:**
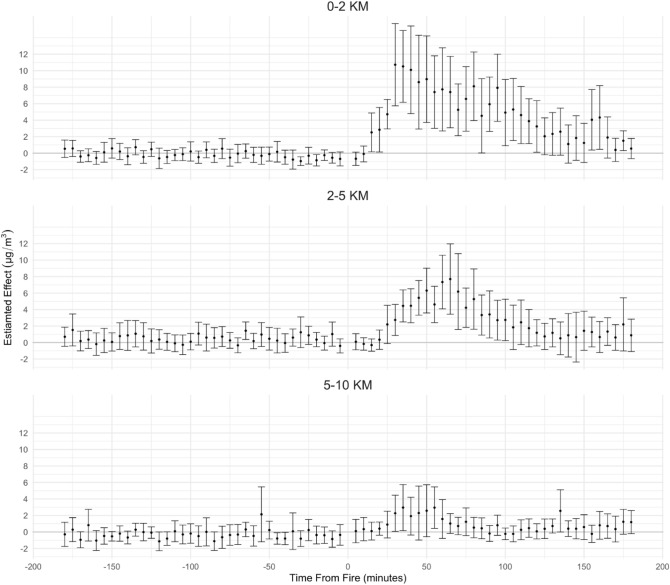
Model-estimated effect of structural fires on PM_2.5_ by distance from fire. Estimates are from models including time and sensor-specific fixed effects and meteorological parameters, with standard errors clustered by sensor. Number of Fires = 21. Number of downwind sensors = 16 (0–2 km), 13 (2–5 km), 11 (5–10 km).

Table 1 evaluates socioeconomic and demographic disparities in census block groups overlapping versus not overlapping the downwind bands. Affected census block groups have statistically significantly lower median incomes and relatively smaller White populations, on average. They also have higher proportions of Black residents and children under 6 years old. There are no significant differences in the proportion of Hispanic residents and elderly residents. Although factors such as local meteorology could affect these results, Fig. 4 shows that the locations of the fires coincide with the neighborhoods of the city with lower incomes and proportionally more Black residents—suggesting that these neighborhoods are more likely to experience fire-associated increases in PM_2.5_ concentrations regardless of the direction of the wind at the time of the fire.

Short-term events may be of particular concern when they affect vulnerable groups. For this reason, we overlay the fire downwind bands with locations of schools and senior centers in Chicago^51,52^. We find that the 5 km area downwind of fires included at least one school for every large fire, affecting approximately 1 in 5 of all Chicago public schools over the one-year period of observation. Similarly, 5 of the city’s 21 senior centers were in the path of the downwind bands at least once.

**Table 1:** Socioeconomic and demographic characteristics of census block groups (CBGs). Results of two-tailed t-test comparing CBGs that fall into at least one of the downwind bands versus those that do not. (df = 2109).

	Downwind Band CBGs		Non-Downwind Band CBGs		T-statistic	P-value
	Mean	SE	Mean	SE		
Median income	64,066	1724	70,745	979	−3.50	< 0.001
Percent White	27.1	1.2	36.6	0.8	−6.31	< 0.001
Percent Black	38.7	1.7	28.2	1.0	5.59	< 0.001
Percent Hispanic	24.8	1.1	27.5	0.8	−1.85	0.06
Percent over 65	13.2	0.4	13.5	0.2	−0.81	0.42
Percent under 6	7.4	0.2	6.5	0.1	3.62	< 0.001
	n = 586		n = 1525			

## Discussion

This study examines the effect of short-term structural fires on PM$_{2.5}$ concentrations. Using a spatially and temporally dense network of real-time PM$_{2.5}$ sensors, we are able to describe the spread and duration of heightened concentrations. We find that, on average, multi-alarm fires contribute up to a 10.7 $\upmu$g/m$^{3}$ increase in PM$_{2.5}$ within 2 km of a fire and a 7.7 $\upmu$g/m$^{3}$ increase 2 to 5 km away from a fire. The heightened concentrations lasted for approximately 2 h after the fire’s start and disproportionately affect neighborhoods with lower incomes, relatively smaller White populations and larger Black populations, and relatively more children under 6 years old.

The observed increases in PM$_{2.5}$ associated with structural fires should be of concern to public health researchers and practitioners for three reasons. First, we observe statistically significant and large increases in PM$_{2.5}$ readings as far as 5 km from the location of fires. The changes in concentrations from a single fires could be much larger, surpassing 20 $\upmu$g/m$^{3}$ in some individual instances, and is likely much larger close to the fire’s source. Considering research that even an additional 1 $\upmu$g/m$^{3}$ of exposure for a day leads to more hospitalizations, our results suggest that structural fires could be meaningfully contributing to adverse health outcomes^9^. Second, the observed increase in PM$_{2.5}$ persists for approximately 2 h on average—a duration that, although short, has been shown to result in negative health effects for vulnerable populations^13,53^. Moreover, multi-alarm fires can last for several hours, suggesting that that the impact on PM$_{2.5}$ likely extends beyond the time periods observed in this study.

Finally, we show that the downwind bands overlapped with areas of relatively larger populations of low-income and Black residents and young children relative to other parts of the city during the year-long period of observation, highlighting an additional burden that should be considered in addition to the existing literature on structural fires’ inequitable effects^27,28^. These increased concentrations may not directly translate into increased emissions burdens, because factors like residential mobility and air quality intrusion will mediate the extent to which increased outdoor concentrations in a particular neighborhood translate into increased exposures amongst its residents. However, prior research suggests that residents of low-income and racially segregated urban neighborhoods spend more time in their home microenvironments relative to residents of other neighborhoods^54,55^ and tend to live in older and less expensive houses that are subject to relatively higher rates of pollution infiltration^56^, likely magnifying disparities in potential health impacts between disadvantaged and advantaged neighborhoods. Furthermore, our work finds that every downwind band affected at least one school—underscoring the potential of real-time monitoring and warning systems to mitigate students’ exposures.

This study is subject to three key limitations. First, our study relies on low-cost sensors, which are subject to error^57^. We thus limit our analysis to PM$_{2.5}$, for which we developed a calibration algorithm to improve accuracy, but further research should investigate the contribution of structural fires to other pollutants. The approach used here could also be extended, with assumptions regarding structure composition and combustion efficiency, to estimate the total mass of structure burned. Second, our results refer to average effects, but we observe considerable variation across fires. We rely on the complete population of fires both to account for confounding factors that may affect any one fire and because there were relatively few sensors downwind of fires: although we examine 21 fires in our study, only 14 had downwind sensors within 5 km, and the distance of those sensors from the fires varied considerably. Nevertheless, the network used in our study is among the largest and most spatially dense low-cost sensor networks deployed in any major city^36^, and offers an important example of how these increasingly common networks can capture changes in PM$_{2.5}$ concentrations associated with local events such as fires. Finally, we also make several simplifying assumptions in our methodology. In particular, the rectangular downwind band is a relatively simple representation of a fire plume and does not consider neighborhood-level wind speed or the urban form of the area impacted by the fire; however, our results are robust to sensitivity analyses using bands representing the fire’s emissions paths of various widths (600 m, 1400 m, 2000 m). Similarly, our meteorological information is limited to that of the two national weather stations in the city and we assume that wind direction is consistent across the metropolitan area and persistent over the 3 hours before and after each fire’s start. Future work should examine how street canyons and other elements of urban form complicate these assumptions.

Nevertheless, our study demonstrates the value of large-scale, low-cost sensor networks in characterizing previously under-monitored urban phenomena. Although an extensive literature documents the disproportionate long-term pollution burdens borne by low-income people and people of color^58–61^, our research offers novel evidence that short-term events follow similarly inequitable patterns. We also highlight the impact of localized events on vulnerable populations such as schoolchildren; future work could build upon these insights to develop real-time and geolocated warning systems that support targeted public health warning systems.

### Supplementary Information

Supplementary Information.

## Data Availability

All data for this study are collected from publicly available sources including Microsoft Project Eclipse^62^, the Chicago Fire Department’s public twitter account^38^, NOAA^40^, the U.S. Census Bureau^50^, and the Chicago Open Data Portal^51,52^. The cleaned analytic data are available from the corresponding author upon request.
