# Supplementary material for: Characterizing the effects of structural fires on fine particulate matter with a dense sensing network
Source: Sci Rep. 2023 Aug 8;13:12862. doi: 10.1038/s41598-023-38392-3 (PMC10409864; doi:10.1038/s41598-023-38392-3)
Supplement: Supplementary file 1 — Supplementary Information. [file 41598_2023_38392_MOESM1_ESM.pdf]

Supplementary Information

Appendix A: Calibration of Eclipse Device

A large literature documents the potential for inaccuracies or imprecision in low-cost optical particle sensors (Maag et al., 2018; Lewis & Edwards, 2016). To address these concerns, we followed previous research in developing calibration functions to adjust readings for potential sources of error (Barkjohn et al., 2021; McFarlane et al., 2021; Zusman et al., 2020). Briefly, 3 additional devices were co-located at each of three EPA regulatory monitoring stations in Chicago (N = 9 Devices total). At each of these sites, the EPA reported hourly average data obtained from Beta Attenuation Monitors (method 731).[1] We applied a machine learning approach to data from May 13th, 2021 until February 28th, 2022 to develop a calibration model as follows.

First, we split the data into training (70%) and test sets (30%). Sensors sample in five-minute intervals, which were then averaged to obtain hourly and daily estimates. We applied a 75% completeness criterion, first excluding hourly data from sensors with fewer than 9 readings in a given hour and then excluding daily data from sensors with fewer than 18 readings in a given day. To account for dependencies in the data—both temporal autocorrelation and the similarities between multiple devices at the same location, reading at the same time—we grouped our data by day before conducting the split. Within the training set, we further used 5-fold cross validation, grouped by day, to train and tune models. We then evaluated model accuracy on our test set. As a check on the generalizability of models from one location and device to a different device at a different location, we also evaluated models through leave-one-station-out cross validation, in which we iteratively excluded one of the three EPA regulatory stations in developing our models and then tested results at the excluded location.

We considered input features including relative humidity (RH), absolute humidity (AH), temperature in Fahrenheit (TempF), pressure (P), gas readings (NO_2, O_3, CO, SO_2) and fine particulate matter ($PM_2.5$). We also included meteorological parameters—wind speed (WS) and wind direction (WD)—obtained from the National Oceanic and Atmospheric Administration weather station at Chicago O'Hare Airport. Given prior evidence of nonlinear relationships with RH (Zusman et al., 2020), we further assessed whether using exponential transformations, b-splines (BS0, BS1, BS2), or interactions with other variables improved the models' fit. To increase robustness to outliers, gas readings were winsorized at the 0.001 and 0.999 percentiles.

We experimented with a variety of possible models; we then focused on developing a smaller subset that performed well on the initial training data. These included linear regression, random forest regression, gradient boosting regression, and categorical boosting regression. Linear regression, random forests, and gradient boosting are among the most common models used for calibration in prior literature (Maag et al., 2018); gradient boosting, in particular, is a leading method for learning problems in which data are noisy, features are heterogenous, and relationships are complex. We also

[1] These stations also recorded 24-hour data on PM2.5 every sixth day using MetOne sequential monitors. In order to ensure adequate sample size as well as comparability between scales of aggregation, we use hourly data for calibration across sites.

considered categorical boosting, a method that mitigates problems of prediction shift—a bias in the residual errors that affects the generalizability of gradient boosting on unseen data (Prokhorenkova et al., 2018).

Table A1: Evaluation of Calibration Models for $PM_{2.5}$. Metrics are calculated following EPA guidelines for performance protocols and metrics (Duvall et al., 2021)

	RMSE	NRMSE	R^2	SD	CV
**EPA Target**	$\leq$ **7**	$\leq$ **30%**	$\geq$ **0.70**	$\leq$ **5**	$\leq$ **30%**
Raw Data	5.3	57%	0.56	1.1	11.2%
5-Fold Cross Validation	3.9	37%	0.62	1.3	12.4%
LOSO Cross Validation	3.4	32%	0.69	1.0	9%
Test Set	2.7	26%	0.70	0.9	7.6%

RMSE = Root Mean Square Error; NRMSE = Normalized Root Mean Square Error
SD = Standard Deviation; CV = Coefficient of Variation; LOSO = Leave-One-Station-Out.

After finding the top regression methods using an initial set of all features, we evaluated all possible input feature combinations to find the best performing model. We then iterated through the regression methods once more to confirm that the top methods remained the best-performing using the new predictors. We investigated additional adjustments to further improve our models. These included scaling, polynomial regression, and hyperparameter tuning; we tested these adjustments using AzureML automated ML model-training jobs. Results were ultimately comparable with models using default hyperparameters. Finally, we selected and evaluated the calibration model with the best overall performance.

Figure A1: Importance Score of Input Features of $PM_{2.5}$ Calibration Model

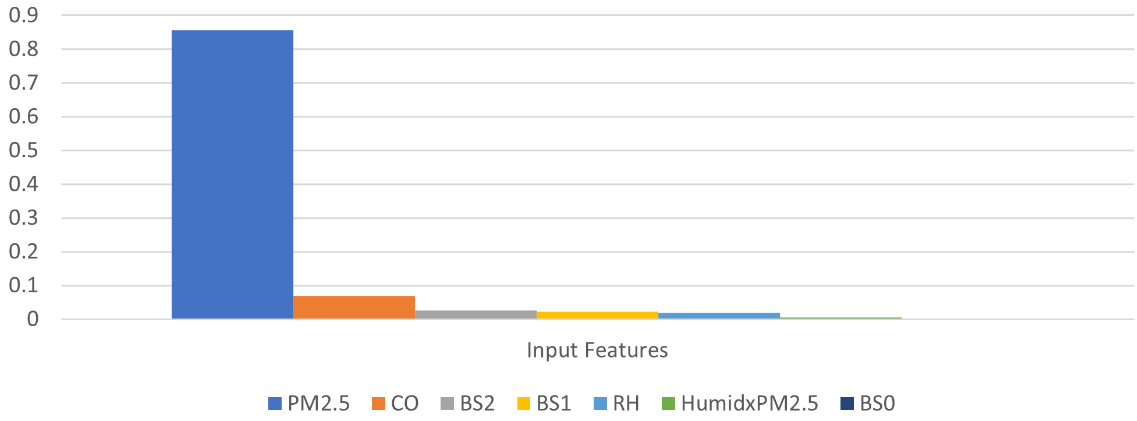

The best-performing model for $PM_{2.5}$ was gradient boosting regression. Table A1 presents the performance metrics for our model. Across metrics, estimates calculated with test set data are consistent with EPA-recommended targets for low-cost sensor use (Duvall et al., 2021). The relatively low root mean square error (RMSE) and normalized root mean square error (NRMSE) as well as acceptable R^2 are evidence that the device produces meaningful signal despite the noise inherent in the usage of low-cost sensors. Also important is the low standard deviation (SD) and coefficient of variation (CV) on the raw data as well as in cross validation or the test set, which suggests that sources of error affect different devices in similar ways. This finding, along with the similar metrics for leave-one-station-out cross validation as on 5-fold cross validation, bolsters

our confidence that an algorithm developed at a given location can generalize to a different device located at a different location.

Because our selected model uses a boosting method, we are able to estimate the relative importance of the included parameters (Figure A1). As expected, the $PM_{2.5}$ reading is the most important parameter in the model; additional features including functions relative humidity are consistent with prior literature (Zusman et al., 2020; Barkjohn et al., 2021; McFarlane et al., 2021). The inclusion of gas readings from the CO sensor provides an additional accuracy boost, consistent with mechanistic understandings of the contribution of combustion-based sources to fine particle pollution. Given these findings, we apply the gradient boosting model to all data used in this study.

Appendix B: Additional Figures and Results for Robustness Checks

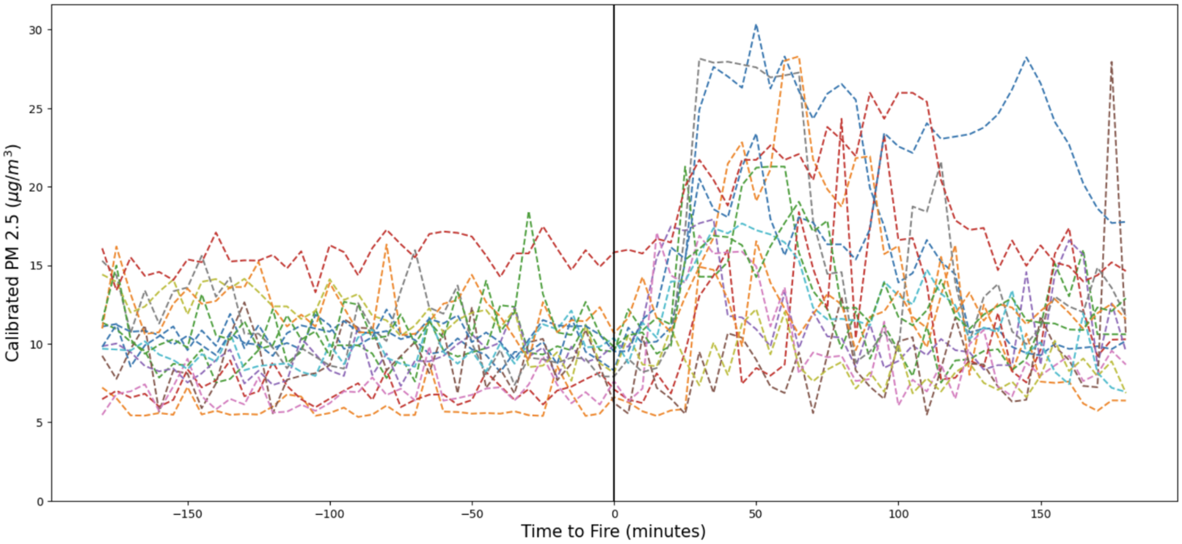

Figure B1: Average PM2.5 readings of downwind sensors (N = 29) for all individual fires with at least 1 sensor within the downwind band (N = 14 fires). The downwind band is defined here as a rectangular band that is 1km in width and 5 km in length from the location of the fire.

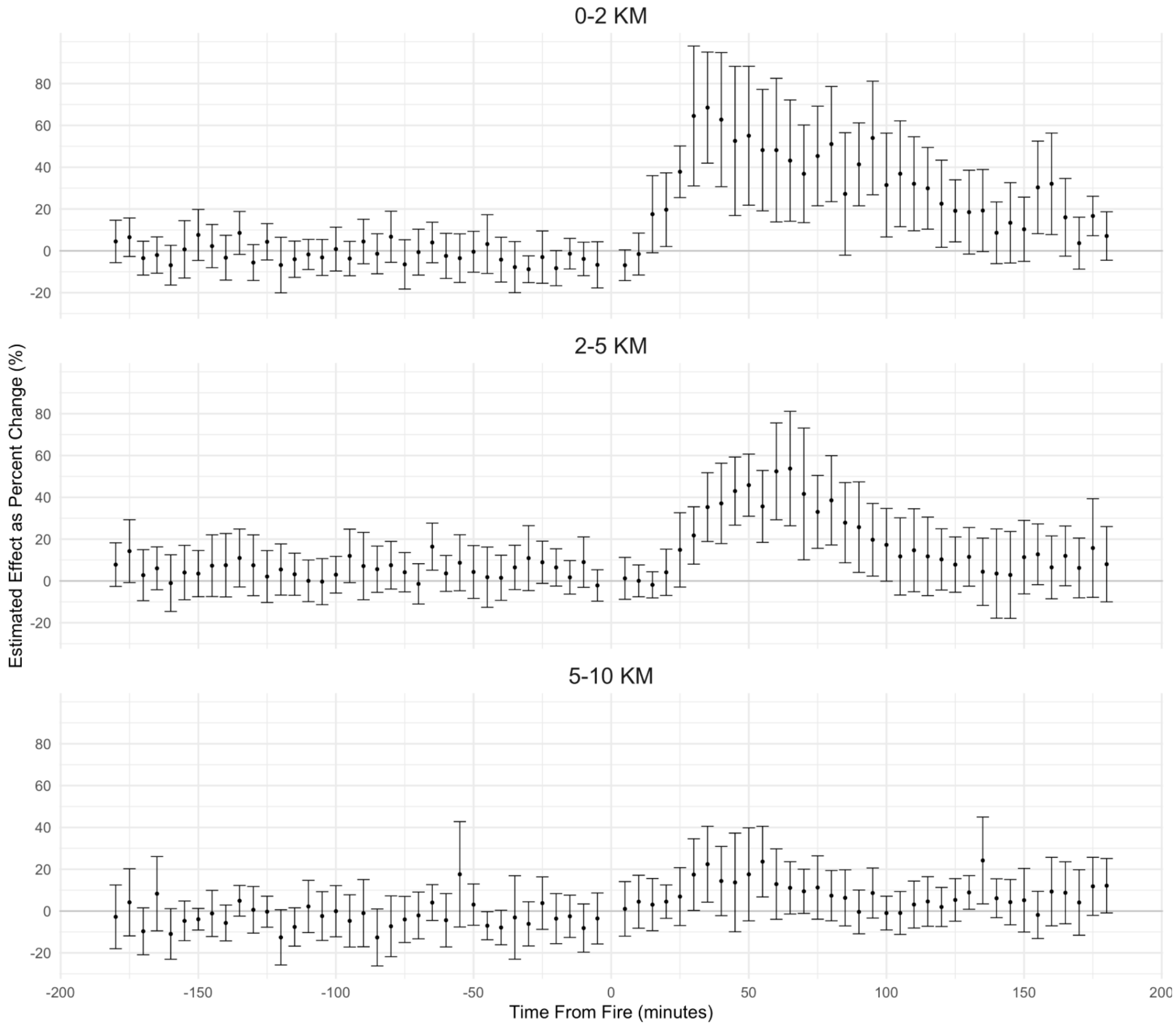

Figure B2: Estimated percent change in PM2.5 levels by distance. Results are from models with logged outcome variables, with treatment groups stratified by the distance of sensors to the fire. Downwind sensors are identified using a rectangular band with 1km width. Model includes time and sensor-specific fixed effects as well as meteorological parameters. Standard errors are clustered at the sensor level. Number of downwind sensors = 16 (0-2km), 13 (2-5km), 11 (5-10km)

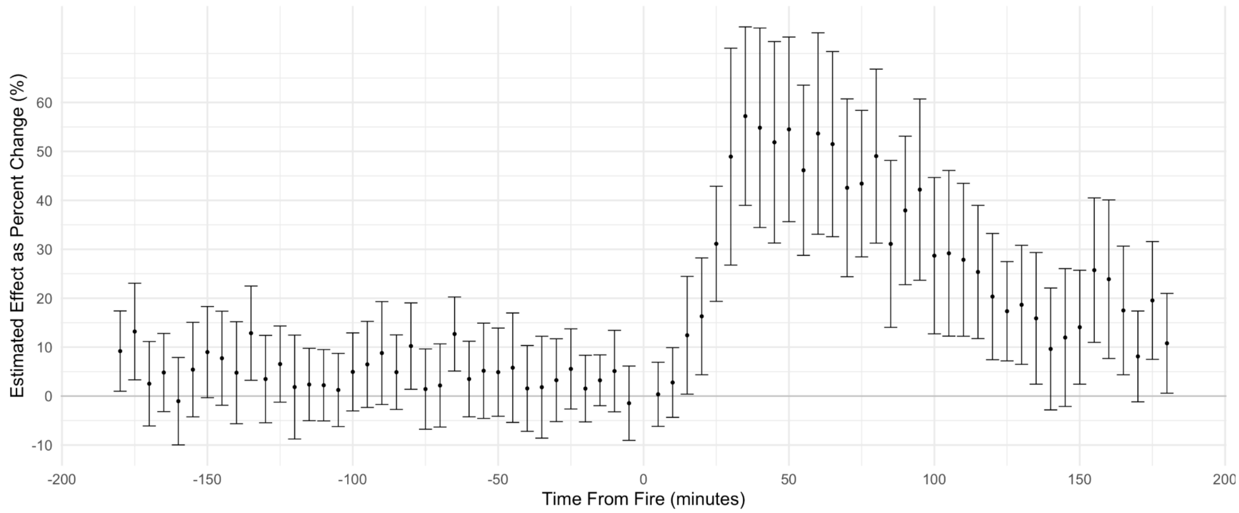

Figure B3: Estimated percent change in PM2.5 Levels. Results are from models with logged outcome variables. All sensors are within 5 km of fires, and downwind sensors are identified using a rectangular band with 1km width. The model includes time and sensor-specific fixed effects and meteorological parameters. Standard errors are clustered at the sensor level. Number of downwind sensors = 29. Number of upwind sensors = 312.

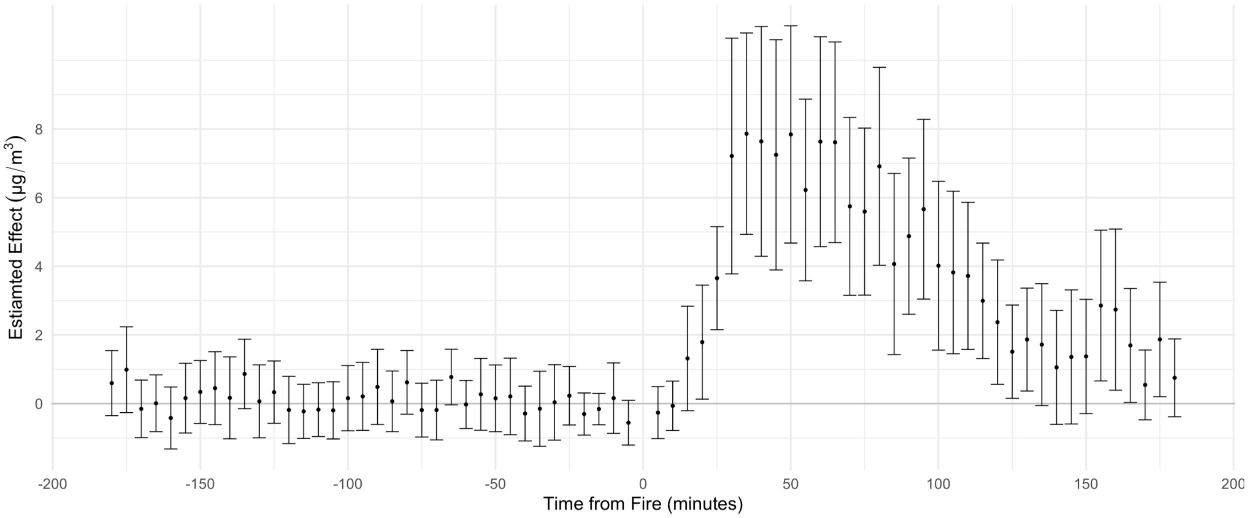

Figure B4: Estimated effect of structural fires on PM2.5 including all non-downwind sensors as upwind sensors. Standard errors are clustered at the sensor level. Estimates are from a model that includes time-, fire-specific and sensor-specific fixed effects and meteorological parameters. Number of Fires = 21. Number of downwind sensors = 29. Number of upwind sensors = 312

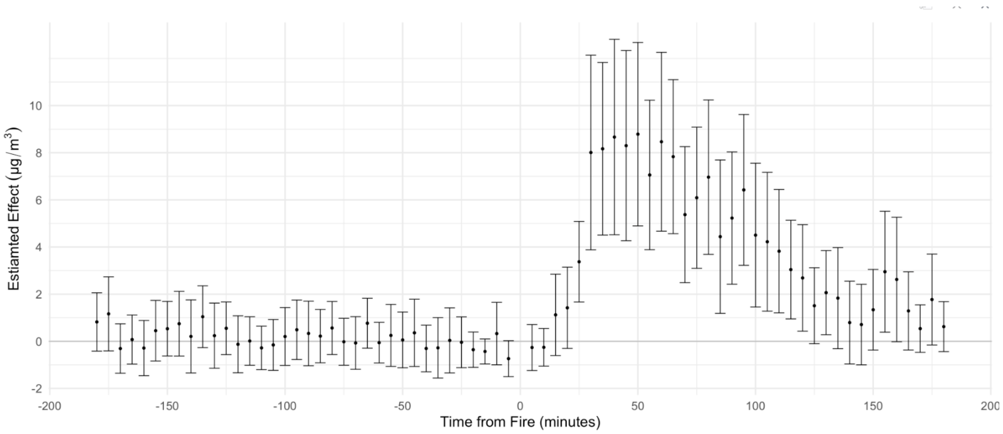

Figure B5: Estimated effect of structural fires on PM2.5 levels with downwind sensors identified using a 600m wide and 5km long downwind band. Upwind sensors are conservatively identified as all sensors within 5 km that do not fall within the downwind band. Standard errors are clustered at the sensor level. Estimates are from a model that includes time, fire-specific and sensor-specific fixed effects, and meteorological parameters. Number of Fires = 21. Number of downwind sensors = 22. Number of upwind sensors = 319

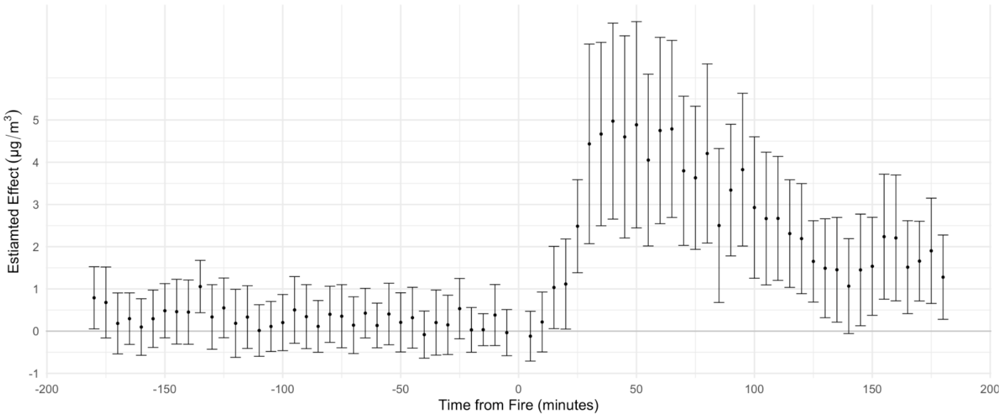

Figure B7: Estimated effect of structural fires on PM2.5 levels with downwind sensors identified using a 2km wide and 5km long downwind band. Upwind sensors include all sensors within 5 km that do not fall within the downwind band. Standard errors are clustered at the sensor level. Estimates are from a model that includes time, fire-specific and sensor-specific fixed effects, and meteorological parameters. Number of Fires = 21. Number of downwind sensors = 49. Number of upwind sensors = 292

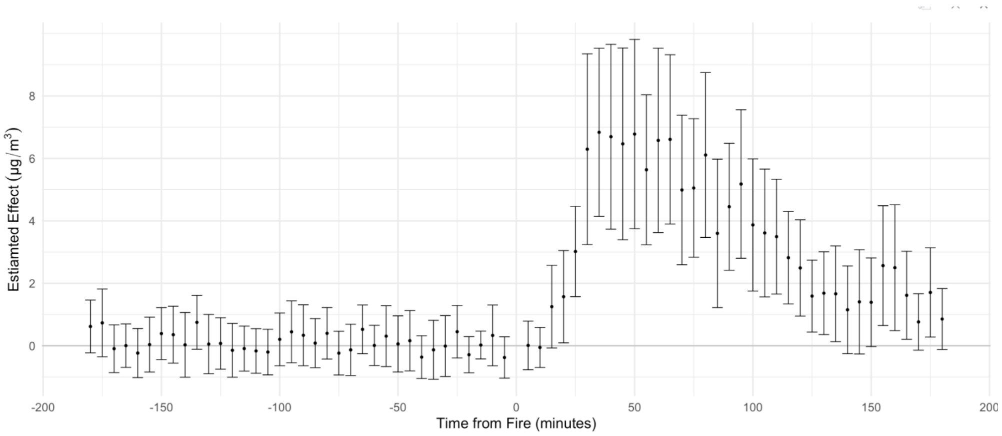

Figure B6: Estimated effect of structural fires on PM2.5 Levels from with downwind sensors identified using 1.4km wide and 5km long downwind band. Upwind sensors are conservatively identified as all sensors within 5 km that do not fall within the downwind band. Standard errors are clustered at the sensor level. Estimates are from a model that includes time, fire-specific and sensor-specific fixed effects, and meteorological parameters. Number of Fires = 21. Number of downwind sensors = 34. Number of upwind sensors = 307

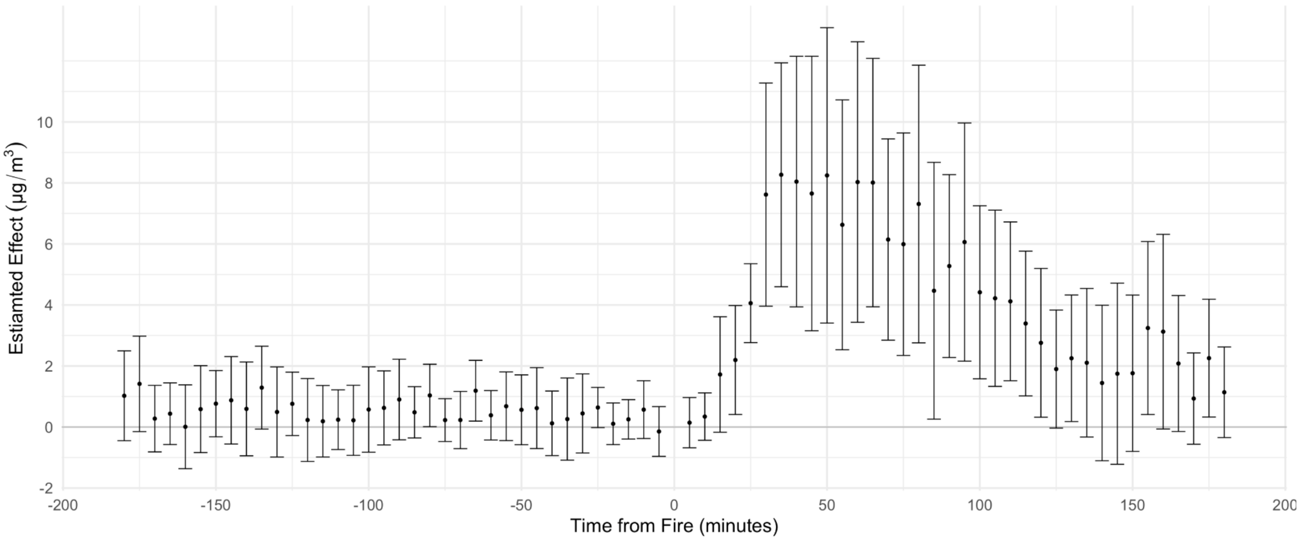

Figure B8: Estimated effect of structural fires on PM2.5 Levels with two-way clustering of standard errors at sensor level and fire level. Downwind sensors are identified using a band with width of 1000m. All sensors are within 5 km of fires. Model includes time- and sensor-specific fixed effects and meteorological parameters. Number of downwind sensors = 29. Number of upwind sensors = 312.

	Downwind Band CBGs		Non-Downwind Band CBGs			
	Mean	SE	Mean	SE	T-statistic	P-value
Median Income	64,426	2076	70,074	932	-2.69	< 0.001
Percent White	26.5	1.4	35.9	0.8	-5.66	< 0.001
Percent Black	39.0	2.0	29.0	0.9	4.86	< 0.001
Percent Hispanic	25.2	1.3	27.1	0.7	-1.19	0.23
Percent Over 65	13.2	0.5	13.5	0.2	-0.53	0.60
Percent Under 6	7.5	0.3	6.5	0.1	3.72	< 0.001
	n = 586		n = 1525			

Table B1: Results of two-tailed t-tests comparing socioeconomic characteristics of census block groups (CBGs) that fall into at least one of the downwind 600 m wide fire bands versus those that do not (df = 2109).

	Downwind Band CBGs		Non-Downwind Band CBGs			
	Mean	SE	Mean	SE	T-statistic	P-value
Median Income	64,522	1522	71,235	1027	-3.75	< 0.001
Percent White	28.0	1.1	37.1	0.9	-6.45	< 0.001
Percent Black	37.1	1.5	28.0	1.0	5.21	< 0.001
Percent Hispanic	25.0	1.0	28.0	0.8	-2.00	0.045
Percent Over 65	13.0	0.3	13.7	0.2	-1.56	0.18
Percent Under 6	7.2	0.2	6.5	0.1	2.74	0.006
	n = 586		n = 1525			

Table B2: Results of two-tailed t-test comparing socioeconomic characteristics of census block groups (CBGs) that fall into at least one of the downwind 1400 m wide fire bands versus those that do not (df = 2109).

References

Barkjohn, K. K., Gantt, B., & Clements, A. L. (2021). Development and application of a united states-wide correction for pm 2.5 data collected with the purpleair sensor. *Atmospheric Measurement Techniques*, *14*, 4617–4637.

Duvall, R., Clements, A., Hagler, G., Kamal, A., Kilaru, V., Goodman, L., Frederick, S., Barkjohn, K., VonWald, I., Greene, D., & Dye, T. (2021). Performance testing protocols, metrics, and target values for fine particulate matter air sensors: Use in ambient, outdoor, fixed sites, non-regulatory supplemental and informational monitoring applications. *US EPA Office of Research and Development*, .

Lewis, A., & Edwards, P. (2016). Validate personal air-pollution sensors. *Nature*, *535*, 29.

Maag, B., Zhou, Z., & Thiele, L. (2018). A survey on sensor calibration in air pollution monitoring deployments. *IEEE Internet of Things Journal*, *5*, 4857–4870.

McFarlane, C., Raheja, G., Malings, C., Appoh, E. K., Hughes, A. F., & Westervelt, D. M. (2021). Application of gaussian mixture regression for the correction of low cost pm2. 5 monitoring data in accra, ghana. *ACS Earth and Space Chemistry*, *5*, 2268–2279.

Prokhorenkova, L., Gusev, G., Vorobev, A., Dorogush, A. V., & Gulin, A. (2018). Catboost:

unbiased boosting with categorical features. *Advances in neural information processing systems*, *31*.

Zusman, M., Schumacher, C. S., Gassett, A. J., Spalt, E. W., Austin, E., Larson, T. V., Carvlin, G., Seto, E., Kaufman, J. D., & Sheppard, L. (2020). Calibration of low-cost particulate matter sensors: Model development for a multi-city epidemiological study. *Environment international*, *134*, 105329.
